# Progression-free or overall… That is the question…

**DOI:** 10.4103/0971-5851.76189

**Published:** 2010

**Authors:** Sudeep Gupta

**Affiliations:** *Editor-in-Chief, Associate Professor of Medical Oncology, Tata Memorial Hospital, India. E-mail: sudeepgupta04@yahoo.com*

Even as this issue reaches you, the US Food and Drug Administration (FDA) would have initiated proceedings to remove the metastatic breast cancer (MBC) label from bevacizumab (Avastin, Genentech-Roche) repertoire of indications.[1] This decision has split the oncology community down the middle with passions aflame on both sides. FDA‘s action is a direct consequence of its Oncology Drugs Advisory Committee’s 12-1 rejection of bevacizumab after reviewing the results of a number of randomized trials of this drug in MBC.

Briefly, bevacizumab gained FDA’s accelerated (and provisional) approval in MBC in 2008 based on the data from the pivotal E2100 trial in which its use, in combination with paclitaxel as first-line treatment in MBC, resulted in a dramatic improvement in progression-free survival (PFS) from 5.9 to 11.8 months.[2] However, the trial was stopped early after a pre-planned interim analysis when 65% of the total events had occurred. At the time of initial approval, FDA required further trials to confirm the magnitude of benefit in PFS and to evaluate the impact on overall survival (OS). With E2100 results, almost everyone expected a home run for bevacizumab. This, surprisingly, did not happen. Two subsequent trials of bevacizumab in combination with docetaxel (AVADO trial)[3] and a range of chemotherapeutic agents (RIBBON 1 trial)[4] failed to show any improvement in OS and only modest improvements (approximately 2–3 months increase in absolute values) in PFS. Importantly, the latter two trials were not stopped early, and therefore, their results were subject to less variability than E2100. A subsequent meta-analysis of all bevacizumab MBC trials, with data on almost 2500 patients, presented at the 2010 Annual ASCO Meeting by Joyce O’Shaughnessy, confirmed that there was a statistically significant 2.5 month improvement in PFS but none, not even a hint, in OS.[5] The FDA in its memo to Genentech stated that modest improvement in PFS and response rates balanced against demonstrated lack of improvement in OS, no proven improvement in symptoms and additional, sometimes life-threatening, toxicity due to bevacizumab, constrained it to recommend removal of the MBC indication for this drug. The FDA has, however, encouraged Genentech to conduct further research to identify subgroups that may benefit preferentially from this drug.

Other critics of bevacizumab point out that robust and reproducible improvements in OS have been repeatedly demonstrated with other agents like trastuzumab (also from the Genentech stable) even when a similar and large fraction of patients crossed over in trials. Moreover, other drugs, like some Poly (ADP-Ribose) Polymerase (PARP) inhibitors, have proven a survival benefit (yet to be reproduced) in much smaller trials and with similar confounding by cross over. Why, they argue, should one lower the bar in a disease like breast cancer that has multiple therapeutic options even in subtypes such as the triple negative one?

The debate has not been one sided. Advocates of bevacizumab, most notably the prominent investigators in Roche-Genentech sponsored bevacizumab MBC trials, have come out in its support. Some of them point out that there may be a natural synergy between paclitaxel and bevacizumab due to their separate and combined anti-angiogenic actions, and thus, the results of E2100 are not an aberration. This has yet to be proven in any clinical or pre-clinical model. Somewhat buying this argument of special affinity, the European Medicines Agency (EMEA) decided to continue its approval for bevacizumab in combination with paclitaxel while withdrawing it in combination with docetaxel and denying it in combination with capecitabine. The advocates also point out that the time lived progression-free may have a value for MBC patients that is independent of OS. Moreover, as pointed out above, OS may be confounded by the large cross-over fraction in many trials. It is also pointed out, not without merit, that it is difficult to prove overall survival benefit in a disease such as MBC which is characterized by relatively long post-progression survival. In this view, MBC is a chronic disorder that requires a series of treatments and bevacizumab fits in this scheme either upfront or later, preferably upfront. There have been emotional public appeals of support from breast cancer patients who have benefited from bevacizumab.

There are important lessons in this story. The most important one is that the opportunity to place an event in some perspective is irreplaceable by any surrogate. All stakeholders, including pharmaceutical companies, patient advocates and regulatory agencies must resist the temptation to jump to conclusions based on compelling but premature data. It is a hard temptation to resist in these feverish times, but resist we must. Another lesson would be to not forget the context while applying standards to different situations. For example, similar data with a new agent may justifiably lead to a different conclusion in a disease like metastatic melanoma that is singularly bereft of meaningful therapeutic options. In this context, the FDA decision on the use of bevacizumab in the first-line setting in ovarian cancer is keenly awaited, where it has shown a similar improvement in PFS in two trials unaccompanied (yet) by improvement in OS.

Although, I am somewhat partial to one side of this debate, I acknowledge that there are valid points on both sides. In this surcharged atmosphere, I cannot help but recall Wadali Brothers’ sublime rendition of the great Sufi poet Bulleh Shah’s “*bullah, ki janan main kon*” (often associated with Rabbi Shergill)… we are all in search of personal truths and there may be no universal ones.

## References

[1] U.s Food and Drug Administration, U.S. Department of Health and Human Services, Safety, MedWatch. Avastin (bevacizumab): Process for Removal of Breast Cancer Indication Begun.

[2] Miller K, Wang M, Gralow J, Dickler M, Cobleigh M, Perez EA (2007). Paclitaxel plus bevacizumab versus paclitaxel alone for metastatic breast cancer. N Engl J Med.

[3] Miles DW, Chan A, Dirix LY, Cortés J, Pivot X, Tomczak P (2010). Phase III study of bevacizumab plus docetaxel compared with placebo plus docetaxel for the first-line treatment of human epidermal growth factor receptor 2-negative metastatic breast cancer. J Clin Oncol.

[4] Robert NJ, Dieras V, Glaspy J, Brufsky A, Bondarenko I, Lipatovet O (2009). RIBBON-1: Randomized double-blind, placebo-controlled, phase III trial of chemotherapy with or without bevacizumab (B) for first-line treatment of HER2- negative locally recurrent or metastatic breast cancer (MBC). Program and abstracts of the 2009 Annual Meeting of the American Society of Clinical Oncology; May 29 - June 2.

[5] O’Shaughnessy J, Miles D, Gray R, Dieras V, Perez EA, Zon R (2010). A meta-analysis of overall survival data from three randomized trials of bevacizumab (BV) and first-line chemotherapy as treatment for patients with metastatic breast cancer (MBC). Program and abstracts of the 2010 Annual Meeting of the American Society of Clinical Oncology.

